# Cerium/diethyldithiocarbamate complex as a novel corrosion inhibitive pigment for AA2024-T3

**DOI:** 10.1038/s41598-020-61946-8

**Published:** 2020-03-19

**Authors:** Iman Mohammadi, Taghi Shahrabi, Mohammad Mahdavian, Mazdak Izadi

**Affiliations:** 1 Department of Materials Engineering, Faculty of Engineering, Tarbiat Modares University, P. O. Box 14115-143, Tehran, Iran; 2 Surface Coatings and Corrosion Department, Institute for Color Science and Technology, Tehran, Iran

**Keywords:** Corrosion, Metals and alloys

## Abstract

In this work, cerium-diethyldithiocarbamate (Ce-DEDTC) complex was synthesized as a novel anti-corrosion pigment. The structure of the synthesized pigment was characterized by employing Fourier transfer infrared spectroscopy, X-ray diffraction, thermogravimetric analysis, inductively coupled plasma optical emission spectroscopy, and ultraviolet-visible spectroscopy. All of the characterization techniques showed that the Ce-DEDTC pigment was successfully produced. The electrochemical tests were used to investigate the subsequence effect of the synthesized complex on the corrosion behavior of the AA2024-T3. AA2024-T3 showed a wide passive range in the presence of the Ce-DEDTC pigment. Scanning electron microscopy, optical microscopy, X-ray photoelectron spectroscopy, and contact angle tests were employed to investigate the effect of the synthesized pigment on aluminum surface properties. The result illustrated that the existence of the Ce-DEDTC complex led to the creation of a thin film on the AA2024-T3 surface, which was significantly inhibited the localized corrosion of the aluminum alloy.

## Introduction

Due to high strength to weight ratio and low density, aluminum, and its alloys have found various industrial applications^1–3^. AA2024-T3 which is the precipitation hardening alloy, has various engineering applications in aeronautics and aerospace industries^4^. S-phase Al_2_MgCu and Al-Cu-Fe-Mn are the main intermetallic compounds in the structure of the 2024-T3 aluminum alloy^4,5^. In the presence of these intermetallic compounds, the substrate is locally degraded, especially in the presence of halide species^5^. The utilization of coatings, especially organic coatings, is a promising method to improve the corrosion performance of aluminum alloy against aggressive solutions^6^. But, it is well-known that the organic coatings alone don’t have the long-term protective behavior against the aggressive ions. Due to the permeation of the oxygen, water, and corrosive ions through the organic coatings, as the main corrosive species for the metallic substrates, the metal is degraded even in the presence of the organic coatings^7^. Therefore, for improving the ability of the organic coatings against the destructive species, the use of other materials is a requirement.

Inhibitive pigments are the main additives used to improve the corrosion protection functioning of the organic coatings in corrosive media. Pigments are utilized in different types and they have different protection mechanisms^8–10^. It is generally believed that the pigments are the fine particle size and insoluble materials used as colloidal dispersions which are added to the organic coatings for various purposes^11^. Providing the color, modifying the applications of the coatings, reducing the cost and improving the performance of the coatings are the main pigment applications^11^. Enhancing the organic coatings corrosion inhibition is the most important application of the pigments. Although it was mentioned that the pigments are the insoluble materials, the corrosion-inhibiting pigments should be somewhat soluble in water^12^.

Chromium-based compounds are the main inorganic pigments, which have the capability to release the corrosion inhibitors providing anti-corrosion performance of the organic coatings. It is well known that these complexes release the hexavalent species in contact with water. In this situation, the metallic substrate passivates, and a thin film is formed by the reaction of the base substrate and the chromium-containing inhibitors. Therefore, the corrosion properties of the metallic substrates are significantly improved by employing the chromium-based pigments^13–15^. Although chromium-based systems still remain as a low-cost and effective source for improving the corrosion properties of the metallic substrates in the aeronautics and aerospace industry, the toxicity and carcinogenic effects of these compounds have begun severe restrictions on their use by international environmental standards^16^.

Due to the limitation of the chromium-based compounds, various pigments have been employed to improve the corrosion performance of metallic substrates^17–21^. As a result, the research works are being sought to find a more benign corrosion prevention technologies based on eco-friendly pigments^12,22–24^. Rare earth metal compounds are suggested as alternative materials for chromates to improve the corrosion properties of the metallic substrates^16,25–31^. Among all of the rare earth compounds, it has been shown that the cerium-based compounds are the most effective materials to provide aluminum alloy protection performance against corrosion^30,32–35^. It has also been shown that organic inhibitors can be engaged as potentially more benign alternatives. In many cases, these compounds need much higher quantities to be operative^36–38^. To decrease the amount of organic inhibitors and also improve the corrosion performance of these species, combination of the rare earth metals and organic ligands can create a multifunctional corrosion inhibition pigment^37,39–42^. It this situation, the mixture of an organic compound and a rare earth metal might provide superior corrosion properties in comparison with the individual constituents at the same concentration^16,34,41–44^.

Due to the presence of the thiol group in the structure of the DEDTC, which also was found in very active corrosion inhibitors, this compound have a potential application to be used as an inexpensive corrosion inhibitor for the AA2024-T3^45–47^. Ferrer *et al*.^48^ used the NaY zeolite particles double-doped with cerium and diethyldithiocarbamate (DEDTC) to improve the corrosion inhabitation of the AA2024-T3 in saline solution. Their results showed that double doping process improved anticorrosive behavior of AA2024-T3 in aqueous saline solution and also suggested potential synergetic effects between Ce and DEDTC.

In the present study, the cerium-diethyldithiocarbamate (Ce-DEDTC) complex was synthesized as a novel inorganic-organic pigment to improve the corrosion protection performance of the 2024-T3 aluminum alloy. For this purpose, a simple blending method was used to synthesize the Ce-DEDTC complex. Also, X-ray diffraction (XRD), thermogravimetric, inductively coupled plasma optical emission spectroscopy (ICP-OES), ultraviolet-visible spectroscopy, and Fourier transformation infrared spectroscopy (FT-IR) analyses were used for characterization of the produced pigment. Potentiodynamic polarization (PDS), and electrochemical impedance spectroscopy (EIS) tests were employed to evaluate the subsequence effect of the synthesized pigments on the electrochemical behavior of the AA2024-T3. To study the surface changes in the presence of the synthesized pigments, scanning electron microscopy equipped with energy dispersive spectroscopy (SEM-EDS), optical microscopy (OM), X-Ray photoelectron spectroscopy (XPS), and water droplet contact angle (CA) tests were employed.

## Materials and methods

### Materials and synthesis procedure

Cerium (III) nitrate hexahydrate (Ce(NO_3_)_3_.6H_2_O), and sodium diethyldithiocarbamate (C_5_H_10_NS_2_Na) were provided from the Merck Co. Deionized water, NaCl, acetone, and 2024-T3 aluminum alloy were supplied from local companies. The chemical composition of the aluminum alloy was: 0.45% Fe, 0.45% Si, 1.24–1.85% Mg, 0.32–0.94% Mn, 3.88–4.96% Cu, and balanced Al. In order to synthesize the Ce-DEDTC pigments, 5 g/lit (22.2 mM) of DEDTC and 6.55 g/lit (15 mM) of Ce(NO_3_)_3_.6H_2_O were added to the deionized water in the unpressurized reactor. After that, the mixture was stirred by a magnetic stirrer at 50 °C for 24 h. Then, the solid particles were collected by a centrifuged (6000 rpm) apparatus and rinsed 3 times with deionized water to ensure about the elimination of the untreated species. For removing the physical absorption of the water molecules, the produced Ce-DEDTC pigment was dried at 60 °C for 12 h.

### Characterization of the synthesized pigments

The thermogravimetric analysis (TGA) was performed using the PerkinElmer apparatus in the nitrogen atmosphere to study the amount of the organic and inorganic compounds in the structure of the synthesized pigments. In this measurement, the temperature range was 25–700 °C, and the heat flow was 10 °C/min. Fourier transformation infrared spectroscopy (FT-IR) (SHIMADZU) in the region of 4000–400 cm^−1^ and X-ray diffraction (XRD) (X’Pert Pro MPD-PW3040/60 with Cu Kα generated at 40 mA and 40 kV) studies were used to assess the chemical composition and crystalline phases of the synthesized pigments, respectively. Furthermore, inductively coupled plasma optical emission spectroscopy (ICP-OES/VISTA-PRO/Varian-Inc) and ultraviolet-visible spectroscopy (UV-vis/OPTIZEN 3220) in the wavenumber of 190–500 nm were employed to study the extent of released inorganic and organic compounds from the hybrid pigment, respectively.

### Electrochemical tests

To investigate the electrochemical behavior of the AA2024-T3 in the presence of synthesized pigment, the plates were cut in the dimensions of 50 × 30 × 20 mm. Then, the metallic substrate mechanically polished by the SiC emery paper from the grade of 220 to 1000. To ensure the complete degreasing of the AA2024-T3 samples, they were ultra-sonicated in acetone for 10 min. Finally, the samples rinsed by the mixture of the water and ethanol and dried by the nitrogen flow. In order to evaluate the effect of synthesized pigments on the electrochemical behavior of the samples, they were immersed in the aqueous extract solution of the Ce-DEDTC pigments. For this purpose, 5 g of the synthesized pigments were added to 1 l of 3.5 wt.% NaCl solution and the mixture was mixed by a magnetic stirrer (500 rpm) for 24 h. The extract solution was provided after the removing of the solid particles using a centrifuge at 6000 rpm. In all of the electrochemical tests, a three electrodes cell was used in which the 1 × 1 cm of the samples was considered to be working electrode and the reminder of the surface isolated by the beeswax, the counter electrode was platinum, and the reference electrode was saturated calomel electrode (SCE). In the potentiodynamic polarization test, the sweep rate and the polarization range were 0.5 mV/s and +250 to −750 mV vs. open circuit potential (OCP), respectively. For the EIS measurements, a 10 mV sinusoidal perturbation signal was used in the frequency range of 10 mHz to 100 kHz. For the EIS tests, 5 points per frequency decade were recorded and the curves were fitted employing Zview software. The EIS experimental tests were repeated three times to ensure about the repeatability of the results.

### Surface studies

To study the surface changes during the immersion of the aluminum alloy in the presence of the synthesized pigments, the specimens cut in the dimension of 1 × 1 cm and prepared according to the preparation procedure which described for the electrochemical tests in the previous section. Then, the samples soaked in the Ce-DEDTC pigments extract solution, and the surface changes were recorded versus the soaking time. The corrosion products chemical composition and morphology were studied by employing the scanning electron microscopy (FEI ESEM Quanta 200) equipped energy dispersive spectroscopy (EDAX EDS Silicon drift 2017). To study the localized corrosion of the specimens, optical microscopy (OM/Olympus) images in various magnifications over soaking times were employed. The X-ray photoelectron spectroscopy (XPS/Specs EA 10 plus) test was used to study the film formation during immersion of samples in the extract solution of the pigments. An OCA 15 plus contact angle device was used to determine the static contact angles of the immersed samples over time.

## Results and discussion

### Characterization of the synthesized pigment

**Figure 1:**
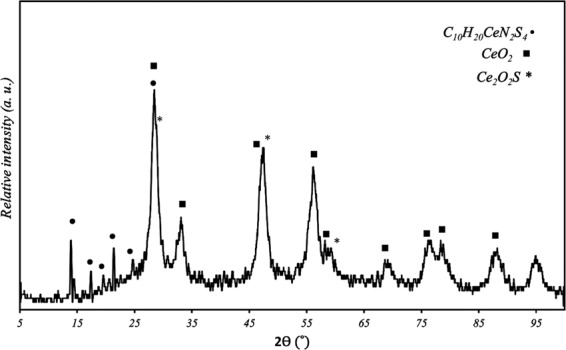
The X-ray diffraction pattern of synthesized Ce-DEDTC pigment.

The XRD pattern of the produced pigment is presented in Fig. 1. Diffraction peaks at 13.46, 17.48, 19.48, 21.28, 24.64 and 28.52 clearly show the presence of the organic-inorganic hybrid complex. Due to the large atomic radius and low electro-negativity of the Ce^3+^ cations as the heavy metal, the sulfur atoms in the structure of the DEDTC shared the isolated electron pairs to form coordination bond^49,50^. This mechanism was studied in various research works and has been showed that the reaction of the DEDTC and heavy metals can lead to the creation of C_10_H_20_MN_2_S_4_ complex (the structure of the complex is shown in the protection mechanism section), where M represents the metallic compound. This result was according to the theoretical analysis and experimental works which were carried out to investigate the structure of the carbamate based complex^50^. The CeO_2_ phase is observed in the XRD spectra of the synthesized pigments, which shows that the cerium oxide has been also formed.

**Figure 2:**
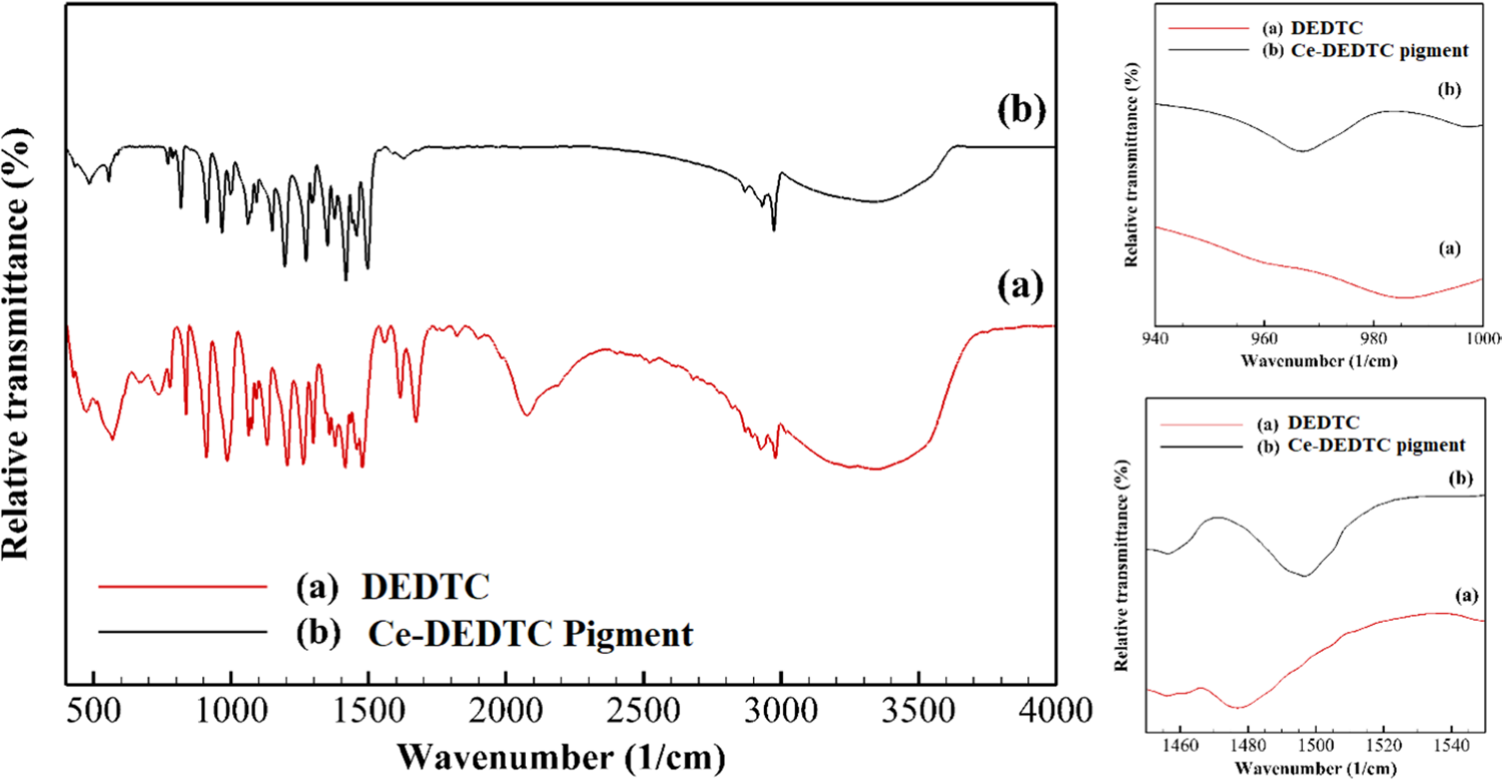
FT-IR spectra of the parent DEDTC and Ce-DEDTC pigment.

The FT-IR spectra of the Ce-DEDTC pigment and parent DEDTC are illustrated in Fig. 2. Based on the structure, the IR spectra of the DEDTC can be interpreted by the vibration bands of the C–S, C–N, C=S, and also the C-H in methyl and methylene groups. The C–N vibration band in NCS_2_^−^ group, appears at the wavenumber of 1450–1550 cm^−1^ ^51^. Furthermore, the vibration band of the C–S in –CSS group and vibration bands of the C-H in the methyl and methylene groups are detected at the region of 950–1000 cm^−1^ and 2800–3000 cm^−1^, respectively^20,51,52^. Based on the literature, the band appears at the regions of 400–500 cm^−1^ is related to the vibration bands of the M-S, which M shows the metallic compound in the structure of the complex^51^. Also, it can be mentioned that the dithiocarbamate group can be coordinated by the symmetric or asymmetric vibration bands which are assigned to the both or one of the sulfur atoms, respectively. It is well known that these frequency modes can be used as the diagnostic factors to determine the monodentate or bidentate mode of the dithiocarbamate^53,54^. The existence of only one strong C–S vibration band can be due to the bidentate coordination of dithiocarbamate. In the other hands, a double peak for the C–S vibration band is anticipated in the case of monodentate coordination^55^. The result of the FT-IR shows that the FT-IR spectrum of the DEDTC consists of a broad double peak in the wavenumber of 950–1000 cm^−1^ which was related to the monodentate stretching vibration mode of the C–S band. Meanwhile, the Ce-DEDTC pigment has an intensive single peak associated with the bidentate stretching mode of the C–S band. Also, it can be observed that the stretching vibration frequency of the C–S band for the parent DEDTC appears at the wavenumber of 985.15 cm^−1^ which decreased to 966.86 cm^−1^ for the Ce-DEDTC complex. Based on the literature, this positive shift in this vibration frequency indicates the formation of chelate^54^.

As can be seen in the FT-IR spectra of the specimens, the stretching vibration frequency of the C–N band in thioureide structure (S_2_C–N stretching mode) for the parent DEDTC and Ce-DEDTC complex are 1477.82 cm^−1^ and 1497.7 cm^−1^, respectively^51^. Also, as seen that the absorption peak value for the Ce-DEDTC complex is higher than that for the parent DEDTC. This result indicates that the increase in the contribution of the thioureide leads to electrons meso-meric drift from the DEDTC moiety towards the metal center. It also can be seen that the M-S peak in FT-IR spectra of the Ce-DEDTC pigment possesses a negative shift by moving to the lower wavenumber which shows the complex formation^51^.

**Figure 3:**
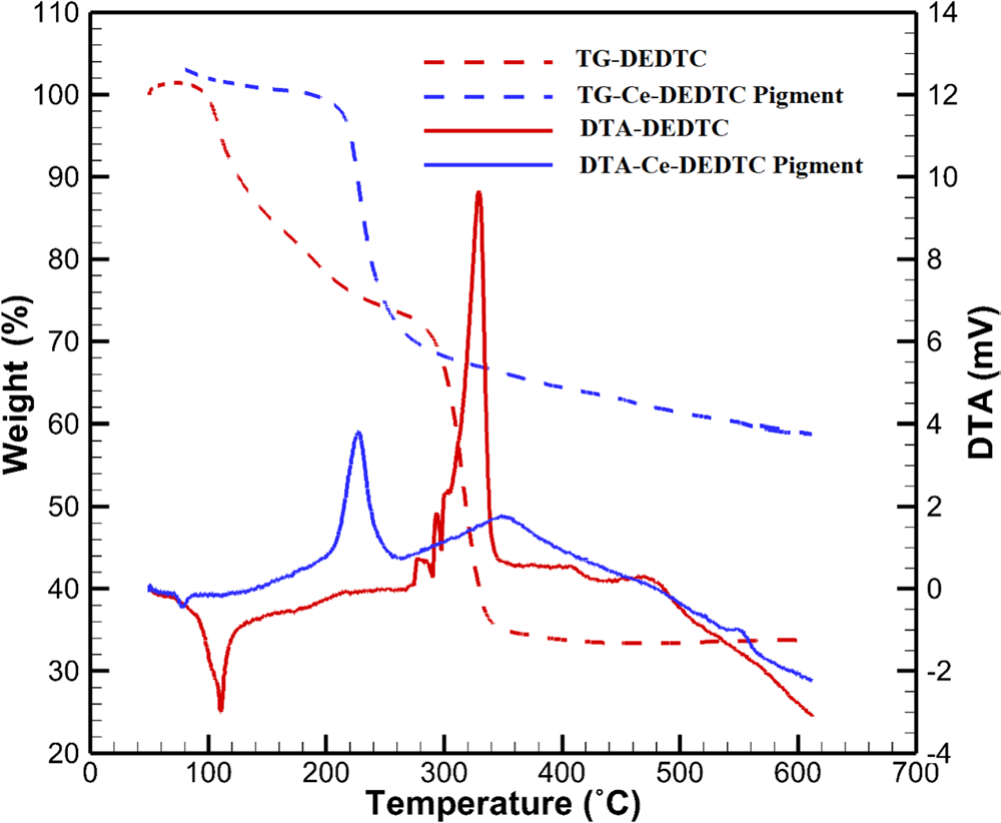
The TG-DSC analysis of the parent DEDTC and Ce-DEDTC pigment.

The TG-DCS test in the dynamic inert atmosphere was used to evaluate the thermal behavior of the Ce-DEDTC pigment. The TG-DCS curves for the Ce-DEDTC complex and parent DEDTC are shown in Fig. 3. It is well known that the mass changes in the materials as a function of temperature can be measured by the TG test^56^. While the DCS measures required heat to keep the sample and reference at the same temperature^57^. The TG-DCS of the samples contains two main peaks. The endothermic peak appears at the temperature range of 80–120 and 80–280 °C for the Ce-DEDTC pigment and DEDTC, respectively. The endothermic peak represents the physically absorbed water in the DEDTC structure^50^. The mass loss at this stage for the Ce-DEDTC complex is about 2%, while this value for the parent DEDTC is about 30%. As seen, the amount of absorbed water in DEDTC is higher than that in Ce-DEDTC pigment. The exothermic peak is appeared in the temperature range of 330 and 350 °C for the DEDTC and Ce-DEDTC pigment, respectively. The weight loss at around 230 °C can be attributed to the dihydroxylation of minor Ce(OH)_3_ phase in the Ce-DEDTC. It is generally believed that this exothermic peak is associated with the thermal decomposition of the sample^50^. Based on the results, the thermal stability of the DEDTC increased by the creation of Ce-DEDTC complex. The total mass loss for the DEDTC is about 73%, which decreased to 42% through bonding with Ce (Ce-DEDTC pigment). This result indicates that the total Ce in the structure of the Ce-DEDTC is more than 29%.

**Figure 4:**
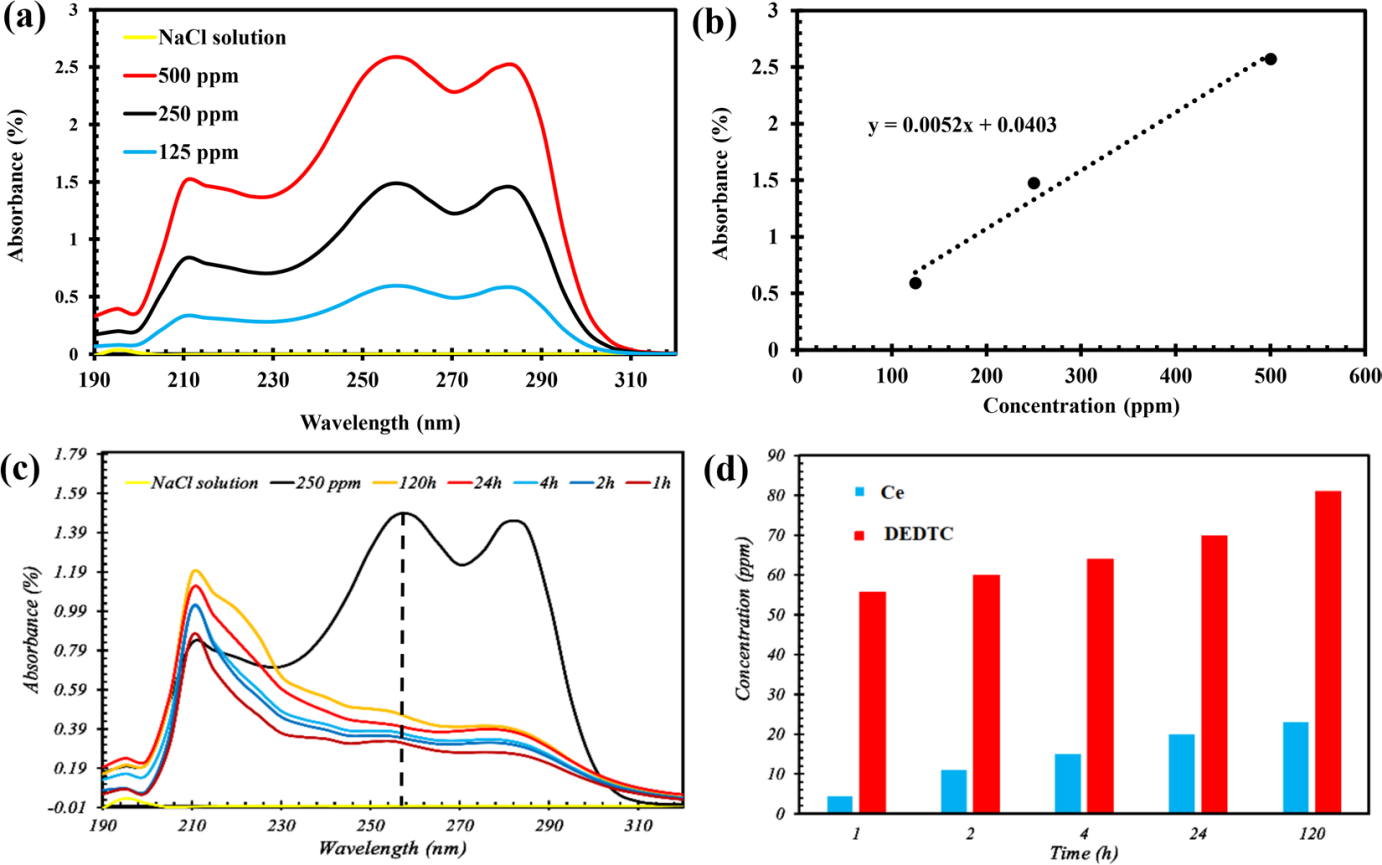
The UV-vis spectra of the 3.5 wt.% NaCl electrolyte, different concentration of the DEDTC and extract solution of the Ce-DEDTC pigment at different dissolution times: (**a**) UV-vis spectra of the 3.5 wt.% NaCl solution at difference concentration of the DEDTC, (**b**) linear regression curve of the DEDTC concentration vs UV-vis absorbance peak at ca. 260 nm extracted from the part (**a**,**c**) UV-vis spectra of the Ce-DEDTC extract solution, and (**d**) the amount on the released DEDTC (measured from the UV-vis result) and Ce (measured by ICP-OES test) from the structure of the Ce-DEDTC complex.

In order to study the partial solubility of the pigment, UV-vis analysis was used in various dissolving times. The UV-vis spectra for the Ce-DEDTC pigments dissolved in 3.5 wt.% NaCl electrolyte versus dissolution time are illustrated in Fig. 4. UV-vis spectra of the Ce-DEDTC pigment extract and DEDTC solution contain 3 main absorption bands. The first absorption band appears at 200–230 nm is associated with the π-π* transient of the sulfur nonbonding electrons^51,58^. The second shoulder centered at 230–270 nm is related to the intra-ligand and π → π* transients found on N-C–S and within S–C=S groups^51,58^. Furthermore, the n → π* electron transients sited on the sulfur atoms appeared at 270–300 nm^51,58^. Based on Fig. 4, compared to the UV-Vis of the parent DEDTC, a negative shift is detected in the location of the first peak of the Ce-DEDTC complex. Also, it can be seen that the intensity of the first peak increased for Ce-DEDTC pigments compared to the DEDTC. This absorption has been intensified upon complex formation with Ce cations. Similar behavior has been reported in literature^59^. Moreover, the location of the second and last peak in UV-Vis spectra of the Ce-DEDTC pigment extract possesses a negative shift in comparison with the parent DEDTC. For studying the amount of released DEDTC from the structure of the Ce-DEDTC pigments, the intensity of the peak at 230–270 nm of the parent DEDTC spectra was investigated and compared with the Ce-DEDTC spectra. For calculating the amount of released DEDTC from the structure of the pigment a linear regression curve showed in Fig. 4b was used. As illustrated in Fig. 4d, the released DEDTC from the structure of the Ce-DEDTC pigment has a time-dependent behavior and the DEDTC released from the structure of the Ce-DEDTC pigment in two steps. At the first region, the amount of the released DEDTC increased rapidly by the dissolution time. It is due to the DEDTC moieties which didn’t react with the Ce ions and are physically adsorbed on the Ce-DEDTC or CeO_2_ pigment surface. At the second region, gradual release of the DEDTC related to the chemical dissociation of Ce-DEDTC complex can be observed.

The ICP-OES test was used to calculate the amount of released Ce ions from the structure of the Ce-DEDTC pigments at difference dissolution times. Figure 4d shows the concentration of the Ce ions which released from the structure of the Ce-DEDTC pigment in 3.5 wt.% NaCl solution versus dissolution time. As can be seen, the released Ce ions have a time-dependence behavior. Also, results indicated that the amount of Ce^3+^ ions in Ce-DEDTC pigment extraction is about 23 ppm after 120 h.

### Electrochemical study

**Figure 5:**
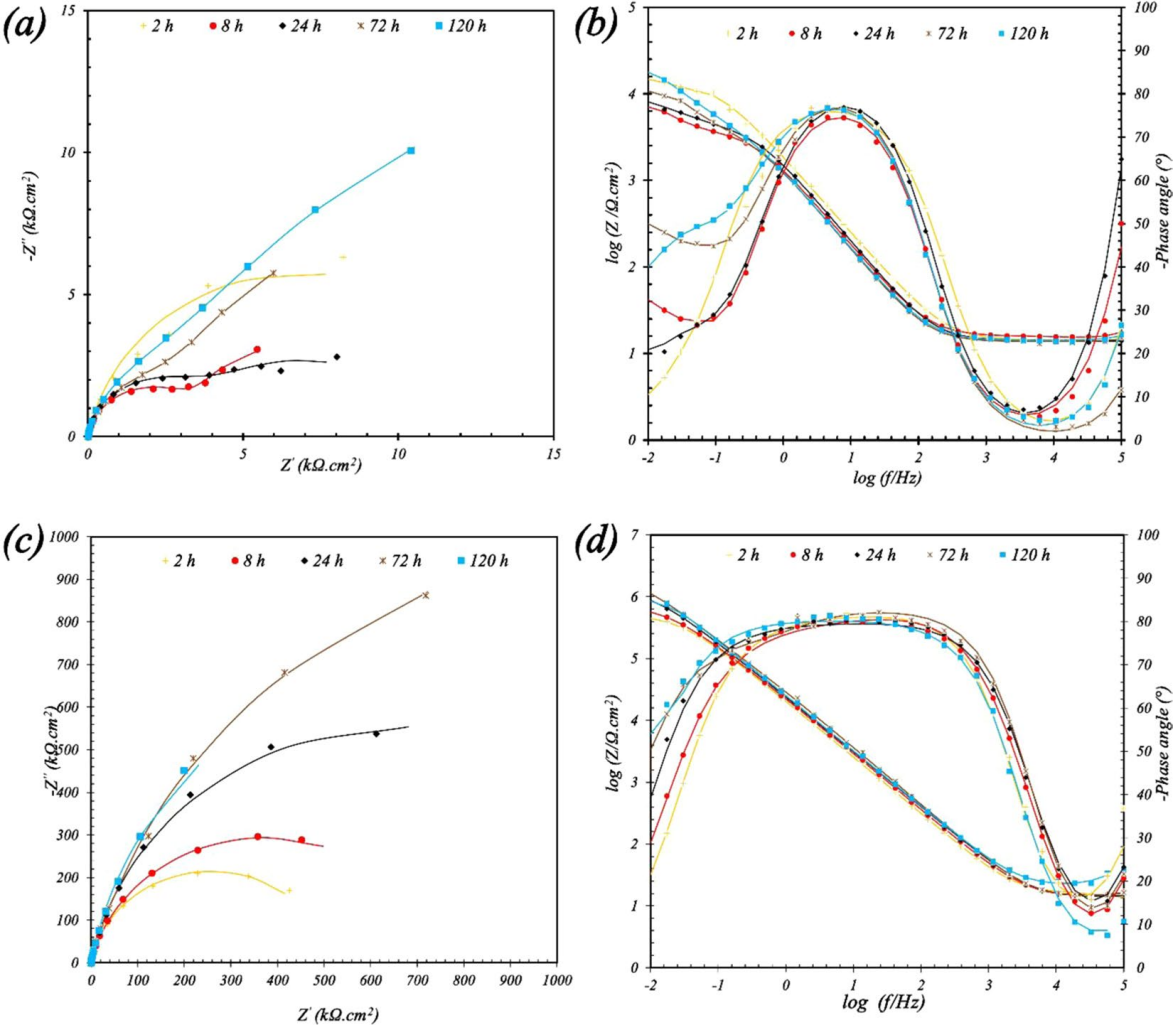
Nyquist and Bode plots for the 2024-T3 aluminum alloy at different immersion time soaked in various solutions: (**a,b**) 3.5 wt.% NaCl electrolyte, and (**c,d**) extract saline solution of the Ce-DEDTC complex.

To investigate the specimen electrochemical behavior, the AA2024-T3 soaked in 3.5 wt. % NaCl electrolyte and extract saline solution of the Ce-DEDTC pigments and the EIS measurements were conducted over the immersion time. The EIS spectra of the blank sample and specimen exposure in the Ce-DEDTC pigment extract solution are illustrated in Fig. 5. As can be observed from Fig. 5, the impedance value for the sample exposed in the Ce-DEDTC pigment extract solution is significantly greater than that for the blank sample in all of the soaking times. Therefore, it can be concluded that the Ce-DEDTC pigments have inhibitive properties against the corrosion of the AA2024-T3. Also, as can be seen from the EIS curves, the impedance value of the blank sample decreased by an increase in soaking time from 2 to 8 h. It can be due to local corrosion occurring on the surface of the specimens. After that, the impedance value of the blank sample increased. The formation of thin film associated with corrosion production is the main reason for this effect. Conversely, the impedance value for the Ce-DEDTC sample increased during the exposure time. This result shows that an inhibitor film formed at the beginning of the immersion times. Also, result indicated that the thickness and also the inhibitive properties of this layer is promoted versus the immersion time.

**Figure 6:**
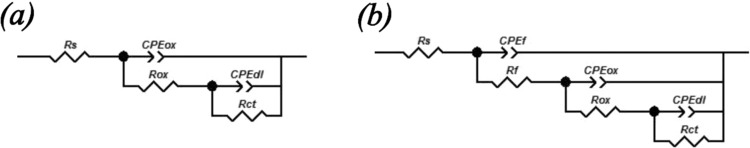
(**a**) Two-time and (**b**) three-time constants, used for fitting the impedance curves of the blank and Ce-DEDTC specimens, respectively.

To investigate the corrosion parameters of the specimen in more detail, the two and three time-constant circuits were employed for fitting the EIS curves of the blank and Ce-DEDTC specimens, respectively (Fig. 6). The equivalent circuit of the blank sample contains a low-frequency time constant associate with the corrosion process on the surface of the specimen and a high-frequency time constant which is associated with the oxide layer naturally created on the AA2024-T3 surface. The equivalent circuit of the Ce-DEDTC contains three-time constants at the low-frequency, middle frequency and high-frequency which are related to the inhibitor film, oxide layer, and double layer, respectively. In these equivalent circuits, the R_s_, R_inh_, R_OX_, and R_ct_ illustrate the solution resistance, the thin film resistance associated with the inhibitor, oxide layer resistance and charge transfer resistance, respectively. Also, CPE_inh_, CPE_ox,_ and CPE_dl_ show the constant phase element of the inhibitor film, oxide layer, and charge transfer of the double layer, respectively. In these circuits, the constant phase element was employed instead of the ideal capacitance. It is associated with this fact that the surface of the samples has a non-ideal behavior, due to the surface heterogeneous^1,2^. To calculate the capacitance of the double layer the following equation (Eq. 1) was used^60^:

1 $${C}_{dl}={{Y}_{0.dl}}^{1/n}{\left(\frac{{R}_{s}{R}_{ct}}{{R}_{s}+{R}_{ct}}\right)}^{(1-n)/n}$$

where n is the exponent of the constant phase element and Y_0_ is admittance. The result of the fitted parameters illustrates in Table 1. The Eq. 2 was used for calculating the R_total_.

2 $${{\rm{R}}}_{{\rm{total}}}={{\rm{R}}}_{{\rm{inh}}}+{{\rm{R}}}_{{\rm{ox}}}+{{\rm{R}}}_{{\rm{ct}}}$$

**Table 1:** The R_total_, R_ct_, and C_dl_ values for the blank and Ce-DEDTC specimens versus the immersion time.

Immersion time (h)	blank*			Ce-DEDTC**		
	R_total_ (kΩ.cm²)	R_ct_ (kΩ.cm²)	C_dl_ (µF/cm²)	R_total_ (kΩ.cm²)	R_ct_ (kΩ.cm²)	C_dl_ (µF/cm²)
2	25	9	185.1	526	442	0.0926
8	61	54	600.6	1108	659	0.0811
24	25	18	275.9	1293	945	0.0286
72	20	13	103.6	2597	2360	0.0198
120	12	6	1932.5	2793	2437	0.0053

*Chi-square is between 0.001–0.005.

**Chi-square is between 0.0004–0.003.

As can be observed from Table 1, the R_total_ for the Ce-DDTS is significantly higher than that for the blank sample. Also, it can be seen that the R_total_ of the Ce-DEDTC increases over the immersion time which shows that the corrosion inhibitor adsorbed on the surface of the specimen.

As can be seen from Table 1, the resistance of the charge transfer for the Ce-DEDTC pigment has a higher value than that for the specimen exposed to neat saline solution. Also, it can be seen that the value of the R_ct_ for the blank sample decrease versus the immersion time. This decrement in R_ct_ values of the blank sample can be due to the destructive effect of the Cl^−^ anions^61^. The bigger R_total_ value and also the R_ct_ increment versus the immersion time of the Ce-DEDTC sample in comparison with the blank sample were associated with the formation of a thin layer during exposure of the sample in extract solution and altering the surface chemical properties of the AA2024-T3. Due to the change in the chemical composition of the surface, the C_dl_ of the Ce-DEDTC sample is much lower than that for the blank sample. Moreover, the C_dl_ for the Ce-DEDTC sample decreases over the immersion time. Conversely, the C_dl_ for the blank sample increases at the prolonged immersion times. The decrement in the C_dl_ values of Ce-DEDTC sample over the immersion time may be associated with the replacing of the water molecules with the corrosion inhibitor presenting in the extract solution of the Ce-DEDTC pigment. Also, considering the capacitance as inversely proportional to the film thickness, the C_dl_ reduction is probably related to an increase in the area covered by the Ce deposition via island growth. Then, a reduction in local dielectric constant is happened in the presence of the Ce-DEDTC pigment^62,63^.

**Figure 7:**
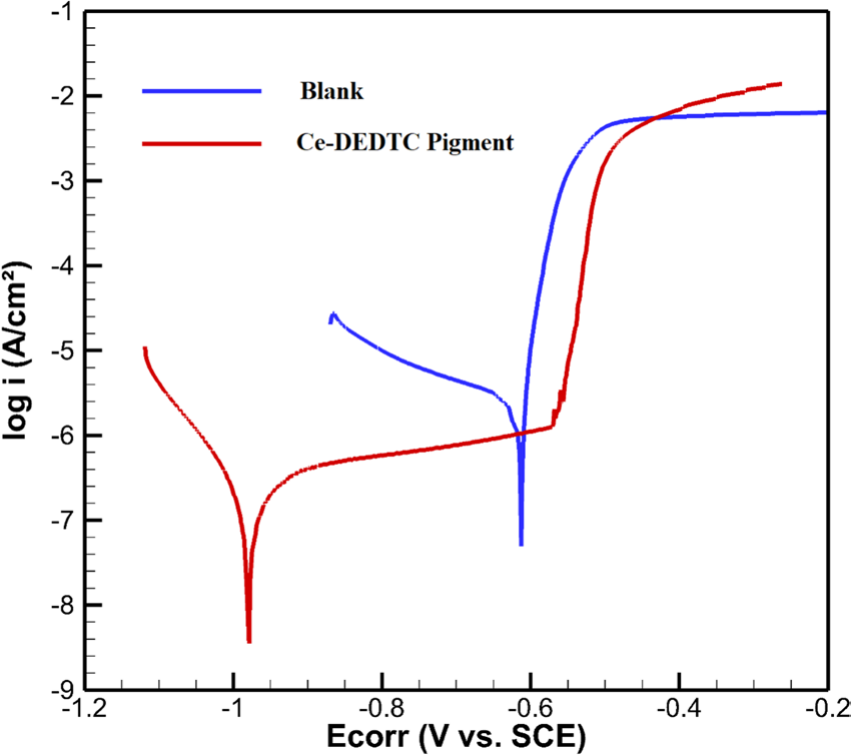
Potentiodynamic polarization curves of the blank and Ce-DEDTC specimens after 120 h of exposure.

The potentiodynamic polarization curves of the blank and Ce-DEDTC specimens after 120 h of exposure are illustrated in Fig. 7. The polarization test was repeated for 5 times and the curves provided in this figure are the curves showing average behavior. The study of the local corrosion is performed as an effective method for evaluating the potentiodynamic polarization parameters. Due to the local dissolution of the bare sample, associated with the intermetallic compound, no passivity range can be seen for the bare sample^5^. Also, to calculate the corrosion current density (*i*_*corr*_) of the specimens, the vertical line crossing the corrosion potential (*E*_*corr*_) and interpolation of the cathodic branch was employed and the extracted results are illustrated in Table 2. As can be observed from Fig. 7 and Table 2, the *E*_*corr*_ achieved more negative value in the presence of the Ce-DDTC pigment. Also, the *i*_*corr*_ of the Ce-DDTC specimen is about 10 times lower than that for the Blank sample. The result illustrates that the passivity range (E_br_-E_corr_) of the Ce-DEDTC is about 427.2 ± 12 mV. Existence of such extensive passive area can be due to the thin film associated with the corrosion inhibitor on the surface of the AA2024-T3. All of these results indicate that the Ce-DEDTC complex can act as an effective anti-corrosion pigment for the 2024-T3 aluminum alloy. The result of the potentiodynamic polarization test is in good agreement with the result of the EIS measurements. Moreover, as can be observed, the current density of the cathodic branch of the polarization curves significantly decreased in the presence of Ce-DEDTC. Therefore, it can be concluded that the Ce-DEDTC acts as a mixed-type with cathodic dominant inhibition suppressing both anodic and cathodic reactions.

**Table 2:** Polarization parameters of the Blank and Ce-DDTC samples after 120 h.

Sample	i_corr_ (A/cm^2^)	E_corr_ (mV vs. SCE)	E_br_ (mV vs. SCE)	E_br_-E_corr_ (mV vs. SCE)
Blank	9.8 ± 0.45 × 10^−7^	−624.3 ± 13	—	—
Ce-DDTC	1.02 ± 0.24 × 10^−7^	−981.3 ± 21	1554.1 ± 12	427.2 ± 12

### Surface study

**Figure 8:**
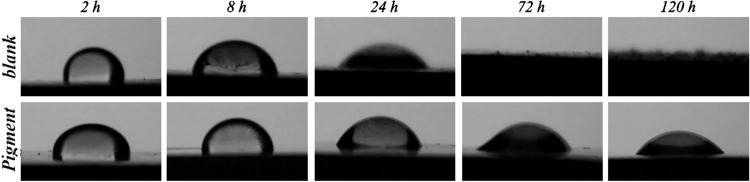
The contact angle images of the distilled water droplet placed on the AA2024-T3 surface soaked in 3.5 wt.% NaCl solution (blank) and extract solution of the Ce-DEDTC pigment (Pigment) after various immersion times.

Figure 8 shows the contact angle test result corresponded to the samples exposed to 3.5 wt.% NaCl electrolyte (blank) and the Ce-DEDTC pigment extract saline solution (Pigment) versus the soaking time. The results of the CA measurement are illustrated in Table 3. In Table 3, the surface energy parameters and work of adhesion (WA) were measured by using Young’s equation (Eq. 3) and Neumann’s equation (Eq. 4)^64^:

3 $${\rm{WA}}={{\rm{\gamma }}}_{{\rm{lv}}}(1+\,\mathrm{Cos}\,{\rm{\theta }})$$

4 $${\rm{WA}}=2{({{\rm{\gamma }}}_{{\rm{lv}}}.{{\rm{\gamma }}}_{{\rm{sv}}})}^{1/2}\exp [\,-\,{\rm{\beta }}{({{\rm{\gamma }}}_{{\rm{lv}}}-{{\rm{\gamma }}}_{{\rm{sv}}})}^{2}]$$

**Table 3:** The values of CA, WA, and γ_sv_ for the AA2024-T3 immersed in 3.5 wt.% NaCl solution (blank) and the Ce-DEDTC pigment extract saline solution (Pigment) after various soaking times.

Specimen	Immersion time (h)	Contact angle (°)	WA (mJ/m^2^)	γ_sv_ (mJ/m^2^)
blank	2	93.5 ± 2%	69.91 ± 2%	26.36 ± 2%
	8	69.8 ± 1%	98.36 ± 1%	41.54 ± 0.02%
	24	31.9 ± 3%	134.29 ± 3%	63.06 ± 0.1%
	72	0.0 ± 0%	145.07 ± 0%	74.12 ± 0%
	120	0.0 ± 0%	145.07 ± 0%	74.12 ± 0%
Pigment	2	98.6 ± 3%	60.36 ± 3%	19.84 ± 1%
	8	95.3 ± 1%	67.53 ± 1%	20.45 ± 1%
	24	67.4 ± 2%	101.56 ± 2%	43.26 ± 0.04%
	72	55.3 ± 2%	114.21 ± 2%	50.42 ± 0.01
	120	45.8 ± 4%	124.05 ± 4%	56.33 ± 1%

In these equations, the value of the β constant is about 0.000125 ± 0.00001 (mJ/m^2^)^2^, γ_lv_ represent the surface tension of water (72 mJ/m^2^), γ_sv_ shows the surface free energy of the specimens, and θ represent the contact angle of water. As can be observed from Table 3, the value of the CA for all of the specimens decreased versus the immersion time. The WA, γ_sv_ of the samples increase over the exposure period. Also, it can be seen that the values of the CA for the Ce-DEDTC pigment are larger than that for the blank sample in all of the soaking times. In addition, the WA and γ_sv_ of the blank sample are larger than that for the Ce-DEDTC pigment specimen. It’s is due to the formation of corrosion product on the AA2024-T3 surface. It is generally believed that the changing in surface chemical composition and roughness are the main reason for the changing in CA of specimens^65^. For the blank sample, as seen in the previous section of this work, the aluminum hydroxide/oxide is fabricated on the specimen surface. Therefore, these changes may lead to changing in roughness as well as the chemical composition of the surface and then decrease the CA of the sample. Furthermore, it was shown that the surface of the sample immersed in the extract solution of the Ce-DEDTC complex is covered by a thin film. So, it can be concluded that the changing in the chemical composition of the 2024-T3 aluminum alloy surface, in the presence of the Ce-DEDTC pigment, lead to increase the CA of the sample. Therefore, the WA and γ_sv_ of the sample significantly decrease in the presence of the Ce-DEDTC pigment extract solution.

**Figure 9:**
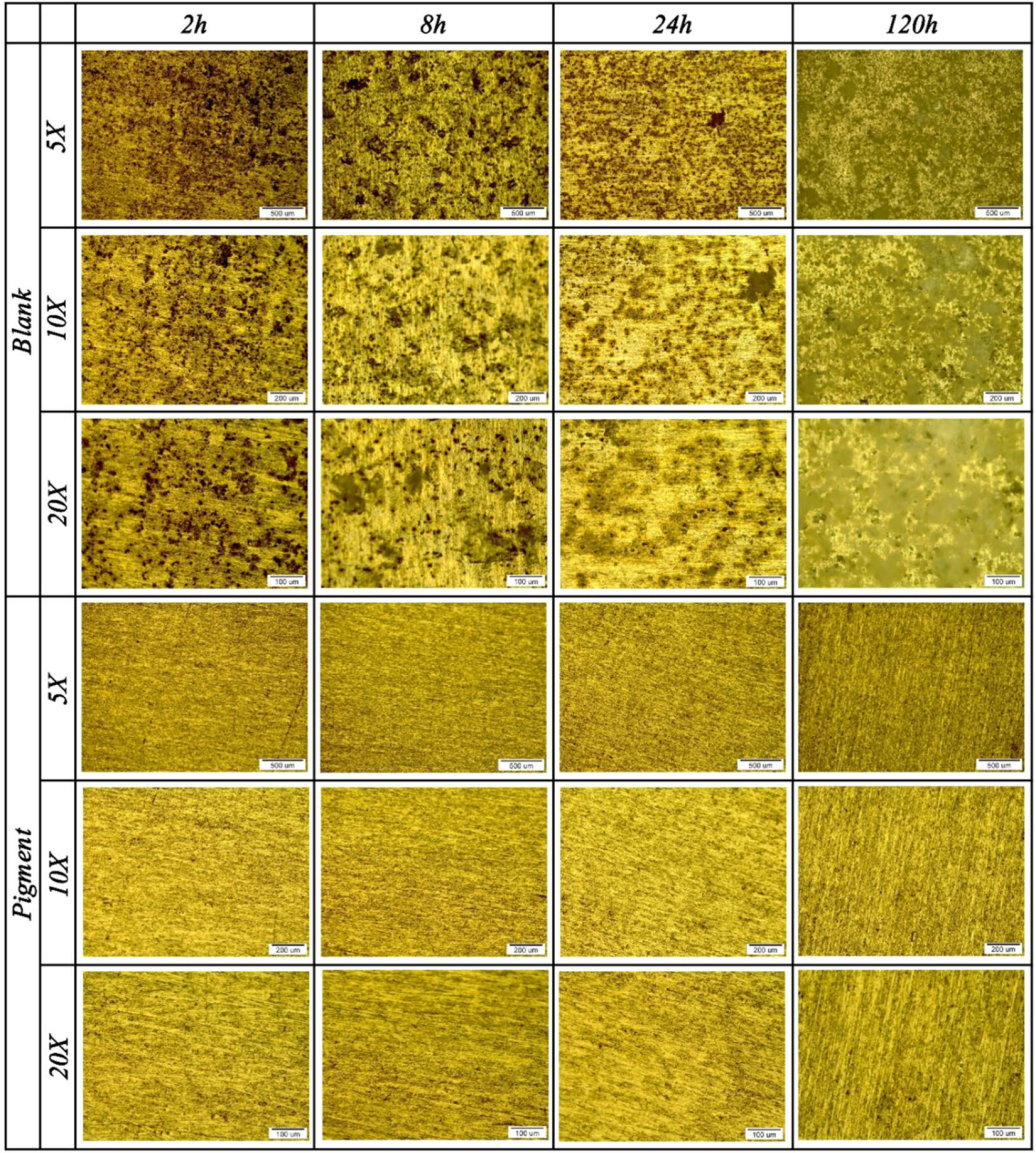
Optical microscopy images for the blank and Ce-DEDTC samples in the various magnifications and immersion times.

In order to investigate the localized corrosion inhibition performance of the specimens, the optical microscopy images were employed. The OM micrographs of the blank and Ce-DEDTC samples at different magnifications and soaking times are demonstrated in Fig. 9. As can be observed, the surface of the blank sample has been degraded by trenching of the intermetallic compounds. This finding is in good agreement with the previous work conducted to study the corrosion behavior of the aluminum alloy in the presence of intermetallic compounds^66–68^. The trenching attack is strengthened at prolonged exposure times. Also, it can be observed that the surface of the blank sample was covered with thick corrosion products at longer immersion times. Also, the big pits can be observed behind the corrosion products. It was associated with the pit coalescence. Based on the OM result, it can be seen that there wasn’t any pit on the surface of the sample soaked in the Ce-DEDTC pigment extract solution even after 120 h. It seems that most of the intermetallic particles suppressed by a surface film. These results are in good accordance with the result of potentiodynamic polarization test and show the better inhibitive performance of the Ce-DEDTC sample against the localized corrosion.

**Figure 10:**
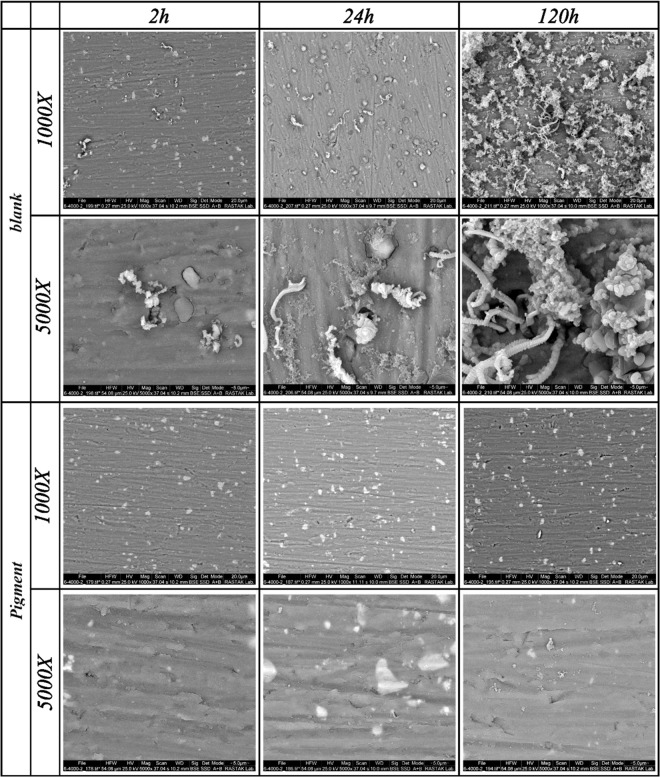
SEM micrographs of the blank and Ce-DEDTC samples at the various immersion times and magnifications.

The SEM-EDS micrographs of the blank and Ce-DEDTC samples in various immersion times and magnifications are shown in Fig. 10. Also, to study the composition of the surface corrosion products formed on the AA2024-T3 in the presence and absence of the Ce-DEDTC extract, the elemental analyses (EDS) was used. The results of the EDS test are illustrated in Table 4. According to the SEM-EDS results the surface of the blank sample contains localized corrosion nearby the intermetallic particles (trenching mechanism). Moreover, the surface of the blank sample is covered by corrosion products at longer times which shows severe corrosion attack on the surface of the specimen. Based on the literature, the intermetallic compounds in the structure of the AA2024-T3 are the main reason for the localizeed corrosion^5^. The increase in the amount of the oxygen on the surface of the blank sample versus the immersion time can be assigned to the aluminum oxide/hydroxide. According to Fig. 10, it seems that the released corrosion inhibitors improve the inhibitive performance of the AA2024-T3 in all of the immersion times. These results indicate the better corrosion properties of the AA2024-T3 in the presence of the Ce-DEDTC complex. Also, the result of the EDS analysis shows that the surface of the Ce-DEDTC specimen contains the C, N, S, and Ce elements as the main compound of the complex showing that the DEDTC species adsorbed on the aluminum surface. Therefore, it seems that a thin film associated with the extract solution of the Ce-DDC pigment is created on the aluminum surface. These results are in good accordance with the EIS test result associated with the thin film formation on the surface of the specimen in the presence of the complex extract solution.

**Table 4:** The composition of corrosion products (atomic %) formed on the surface of the 2024-T3 aluminum alloy soaked in the 3.5 wt.% NaCl solution and Ce-DEDTC extract solution vs. the immersion time.

Specimen	Immersion time (h)	Al	Cu	Mg	O	Ce	C	N	S
Blank	2	82.63	3.77	2.96	10.64	—	—	—	—
	24	68.24	2.63	1.93	27.2	—	—	—	—
	120	55.16	2.63	1.01	49.8	—	—	—	—
Ce-DEDTC	2	89.99	2.62	3.11	1.31	0.15	0.1	0.08	2.64
	24	88.17	3.07	3.14	1.95	0.21	0.15	0.18	3.13
	120	89.85	2.69	3.18	1.31	0.15	0.1	0.08	2.64

**Figure 11:**
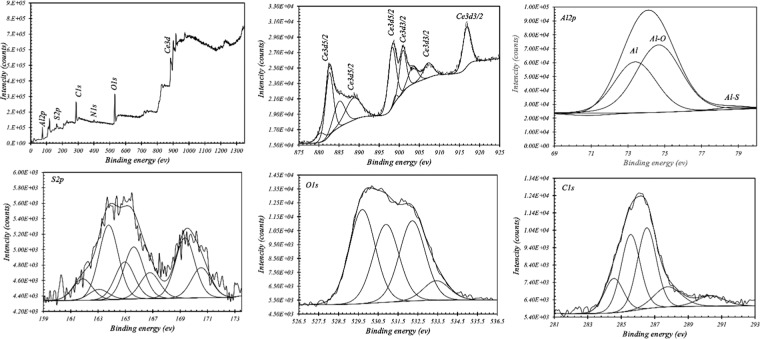
The XPS survey and high-resolution spectra of Ce_3_d, Al_2_p, S_2_p, C_1_s, and O_1_s.

The XPS analysis was used for studying the chelate formation in the presence of the Ce and DEDTC in the extract solution. In order to do that, the aluminum plate immersed in extract solution of the Ce-DEDTC pigment and the XPS spectra was provided from the surface of the specimen after 120 h. The XPS survey and also the high-resolution spectra of Ce_3_d, Al_2_p, S_2_p, C_1_s, and O_1_s are presented in Fig. 11. The deconvolution of high-resolution spectra was conducted to study the potential chemical bonds between the species. The high-resolution spectra of the Ce_3_d show the peaks around the 899.01, 900.9, 903.9, 907.21 and 916.82 eV representing the spin-orbit states of the Ce_3_d_3/2_ assigned to the 3d104f1 electronic state of Ce^3+^ ions^69–73^. Also, the 3d104f0 spin-orbit state attributed to the Ce^4+^ appeared at the 880.41, 882.6, 885.09, 888.61, and 898.22 eV^69–73^. The O1s contains 4 main peaks at around of 529.7, 530.9, 532.22 and 533.43 eV. The peaks at 529.7 eV and 530.9 eV are related to the CeO_2_ and Ce_2_O_3_, respectively^22,74,75^. The peaks at 532.22 eV and 533.43 eV are due to the Al-O bonded and Ce(OH)_3_, respectively^22,74,75^. The high-resolution spectra of the Al2p contain 3 main peaks at binding energy of 72.8, 75.1 and 78.23 eV assigned to Al, Al-O, and Al-S, respectively^22,76^. C 1 s peak can be deconvoluted into five species, the peak corresponding to the C-C/C-H bond with hybridization of sp2 (284.56 eV)^77–80^, the peak at the binding energy of 285.56 eV is associated with the covalent bond of C–S^79,81^, and the sp2 C–N bond appears at 286.53 eV^77–80^. Also, based on the literature, the peaks at the binding energy of 287.78 and 290.35 eV could be due to the formation of S–C–N and S–C–S bonds on the aluminum substrate surface, respectively^82^.

As presented in Fig. 11, the S2p deconvoluted into four peaks at 161.88, 163.77, 165.61, and 169.35 eV. The other peaks are satellite. The peaks around the 163.77 and 165.61 eV are assigned to the sulfur (C–S/S–S) in its elemental state in the structure of the DEDTC^81,83–85^. The peaks around the 169.35 and 161.88 eV can be due to the interaction of the thiol group with the metallic compound^83,84^. Therefore, it seems that the peak around the 161.88 and 169.35 eV can be associated with the C–S–Al^86^ and CeO_2−x_S_x_^87^, which shows the mechanism of the adsorption of corrosion inhibitors on the AA2024-T3 surface.

### Protection mechanism

**Figure 12:**
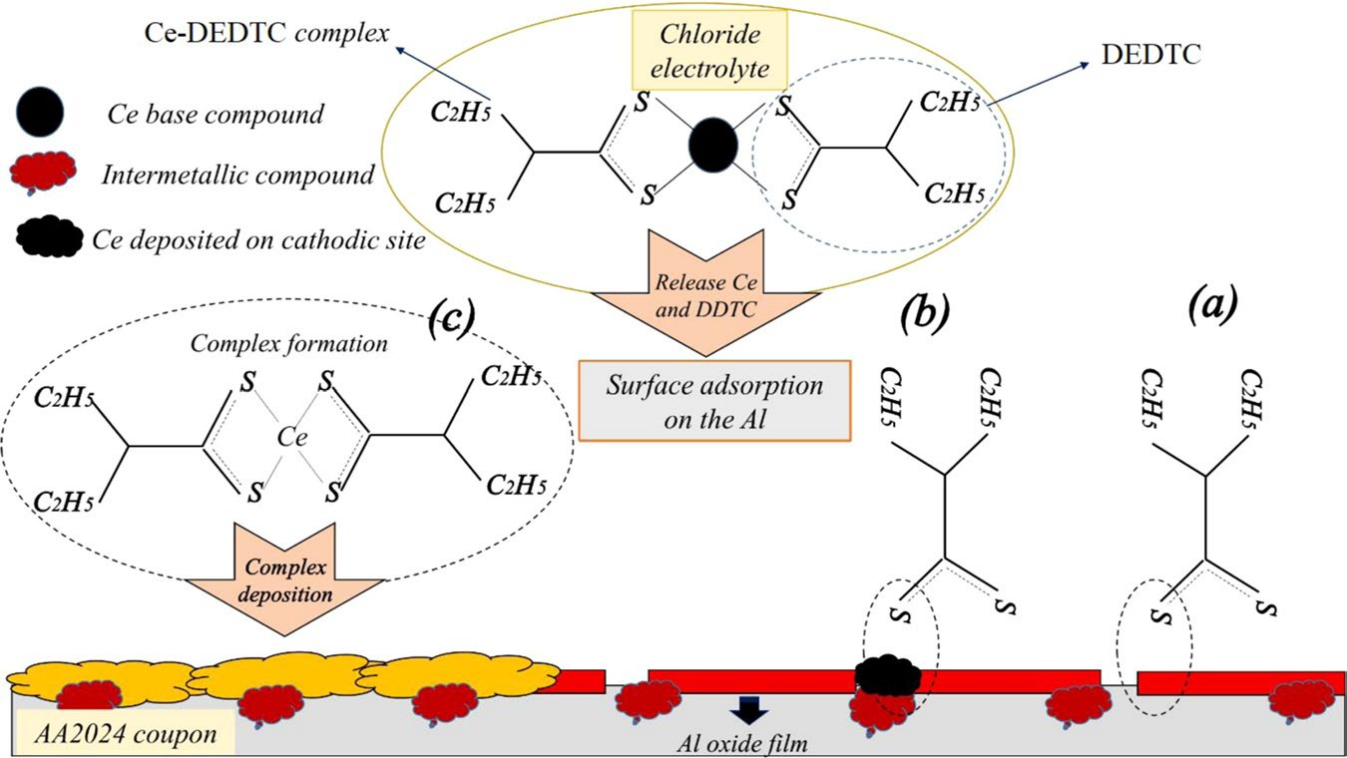
The schematic illustration of different potential mechanisms which improve the inhibition properties of the AA2024-T3 in 3.5 wt.% NaCl electrolyte in the existence of Ce-DEDTC complex.

The schematic illustration of different possible mechanisms which improve the inhibition behavior of the AA2024-T3 is presented in Fig. 12. Based on the characterization results, the synthesized Ce-DEDTC pigment contained the DEDTC and Ce species. Also, it was shown that the Ce ions and DEDTC molecules released from the Ce-DEDTC pigment structure due to its partial dissolution in the saline solution. Therefore, in the presence of the Ce and DEDTC species in the extract solution of the Ce-DEDTC pigment, three main mechanisms can be activated to improve the corrosion behavior of the AA2024-T3. The first mechanism is based on the fact that the thiol sites in the structure of DEDTC can be interacted with the Al^3+^ cations or surface Al oxide film (Fig. 12a). This mechanism leads to deposition of a protective thin film on the active corrosion regions of Al surface. The presence of Al-S bond in XPS result (Fig. 10) proved this mechanism. This mechanism is in good agreement with the result of Forsyth et. al. about the formation of a mixed metal organic species on the surface of the metallic substrate in the condition of presence of cerium salicylate compound in saline solution^39^. They have shown that the physical adsorption of the complex is performed, when the mentioned compound was used for improving the anti-corrosive behavior of the metallic substrate in corrosive media^39^. The second mechanism is based on this fact that the Ce ions which released from the structure of the Ce-DEDTC pigment, due to cathodic performance, deposited on the cathodic sites of the substrate. After that, the thiol group in the DEDTC structure is bonded with the deposited structure (Fig. 12b). The presence of the CeO_2−x_S_x_ phase in XPS result (Fig. 10) confirmed this mechanism. Moreover, the presence of the CeO_2_, Ce_2_O_3_ and Ce(OH)_3_ in the XPS results (Fig. 10) can confirm the deposition of the Ce derivate on the surface of the AA2024-T3. Ferrer et. al. confirmed the cathodic performance of double-doped zeolites with Ce cations and DEDTC molecules for AA2024-T3. Also, their results demonstrated that the Ce-DEDTC complex was created on the intermetallic compounds, when the corrosive media contained these species^48^. The final mechanism is based on the chelate formation (Fig. 12c). The released Ce ions and DEDTC molecules in solution reform complex and deposited on the surface of the aluminum. Based on these results, it could be concluded that the complex physically and chemically adsorbed on the aluminum surface. Therefore, the corrosion behavior of the AA2024-T3 significantly enhanced in the existence of the Ce-DEDTC complex. The results of this work are in good agreement with the previous works conducted to performed the corrosion properties of the metallic substrate in corrosive media in the presence of the Ce-Organic complex. Where, the absorption of the inhibitor via the organic component in the first instance to form a metal–organic bond, followed by the formation of a mixed Ce-organic compound-metal substrate leading to the mixed inhibition^39,40^.

## Conclusion

In this work, the Ce-DEDTC complex was synthesized as a novel anti-corrosion pigment. Also, the subsequent effect of this pigment on the corrosion behavior and also surface properties of the AA2024-T3 was investigated and the following main conclusions were obtained:

- The XRD, FT-IR, TG, ICP-OES, and ICP analyzing methods were used to characterize the produced pigment. The result showed that the pigments successfully were synthesized. Also, the result indicated that the total released Ce and DEDTC species from the 3.5 wt.% NaCl solution contained 5 g/L Ce-DEDTC complex after 120 h were about 23 ppm (0.16 mM) and 70 ppm (0.4 mM), respectively.
- The electrochemical behavior of the 2024-T3 aluminum alloy in the existence of the Ce-DEDTC complex was investigated by EIS and PDS tests. The result indicated that the inhibition performance of the aluminum alloy significantly enhanced in the presence of the Ce-DEDTC pigments. Also, the thin film formation was considered as the main protection mechanism of the Ce-DEDTC pigment. The impedance of the AA2024-T3 was about 100 times larger than that for the blank sample. Also, a wide passivation range was observed in the existence of the Ce-DEDTC pigment extract solution.
- The SEM, EDS, OM, XPS, and CA tests were employed to investigate the subsequent effect of the Ce-DEDTC pigments on the AA2024-T3 surface properties. The results indicated that the resistance of the AA2024-T3 against the pit formation significantly enhanced in the presence of the Ce-DEDTC pigment. Moreover, the results showed that a thin film is created on the AA2024-T3 surface in the presence of the Ce-DEDTC pigment extract.

## Data Availability

The dataset generated or analyzed during the current study are available from the corresponding authors for two years after publication.
